# Anatomic Locations and Metastatic Risk in Prostate Cancer in African Men: Insights From an African Cohort

**DOI:** 10.7759/cureus.62393

**Published:** 2024-06-14

**Authors:** George Asafu Adjaye Frimpong, Evans Aboagye, Osei Owusu-Afriyie, Diane Owusu-Afriyie, Isaac O Antwi, Bernard D Akpaloo, Emmanuel Asante

**Affiliations:** 1 Radiology, Kwame Nkrumah University of Science and Technology, Kumasi, GHA; 2 Radiology, Spectra Health Imaging and Interventional Radiology, Kumasi, GHA; 3 Research and Development, Spectra Health Imaging and Interventional Radiology, Kumasi, GHA; 4 Pathology, Kwame Nkrumah University of Science and Technology, Kumasi, GHA; 5 Radiology, 37 Military Hospital, Accra, GHA; 6 Urology, Tafo Government Hospital, Kumasi, GHA; 7 Radiation Oncology, Komfo Anokye Teaching Hospital, Kumasi, GHA

**Keywords:** zonal predispositions, lesions, cancer, tumour, prostate, metastasis

## Abstract

Background: There is significant variability in the pathogenetic characteristics of prostate cancer (PCa) across different anatomical zones. This study aims to understand the metastatic risk associated with these zonal predispositions among African men.

Methods: This hospital-based retrospective observational study included 120 biopsy-confirmed PCa patients examined between 2019 and 2023. Data on cancer history, sociodemographic, and clinical characteristics were collected from medical records. A logistic regression model was used to identify predictors of metastasis.

Results: The majority of PCa lesions were found in the left (60.0%) and right peripheral zones (55.8%), followed by the left (42.5%) and right transitional zones (41.7%). Lesions in the anterior fibromuscular stroma (crude odds ratio (cOR): 3.27, 95% confidence interval (CI): 1.13-9.47; p = 0.029), central gland (cOR: 5.38, 95% CI: 1.40-20.60; p = 0.014), and diffuse infiltration involving whole gland (cOR: 6.78, 95% CI: 1.17-30.07; p = 0.032) were associated with significantly increased odds of metastasis. Lesions in the anterior fibromuscular stroma were a marginally independent predictor of metastasis (adjusted odds ratio (aOR): 28.14, 95% CI: 0.96-822.46; p = 0.053).

Conclusions: This study underscores the variability in metastatic risk of PCa lesions across different anatomical zones in African men. Lesions in the anterior fibromuscular stroma, central gland, and diffuse infiltration involving the whole gland have higher odds of metastasis. These findings highlight the need for targeted diagnostic and therapeutic strategies based on lesion localization to improve PCa management in this population.

## Introduction

Prostate cancer (PCa) stands as the most prevalent urological cancer, comprising one out of every 14 diagnosed cancer cases globally [1-3]. Among men, PCa ranks as the second leading cause of cancer mortality, following lung cancer [2].

About four decades ago, McNeal introduced the indispensable concept of the zonal anatomy of the prostate, which divided it into peripheral (PZ), transitional (TZ), and central zones (CZ) [4,5]. Several studies over time have shown that each zone has variable PCa incidence, prognosis, and outcomes, with differences in histology and profiling, highlighting their potential role in tumor aggressiveness [6,7].

The advent of magnetic resonance imaging (MRI) and other novel imaging modalities allowed the localization of cancers within the prostate gland, opening the possibility of adding to clinical variables that predict cancer aggressiveness [8]. Currently, there is increasing recognition of the importance of determining the imaging characteristics of the TZ and CZ, as a significant proportion of prostate tumors (up to 30%) originate outside the peripheral zone [6]. Recent advancements, particularly in prostate MRI, have shed light on the unique imaging features of these zones, leading to improved cancer detection and characterization [6,9].

Several studies have indicated a higher risk of metastases associated with a higher Gleason grade and prostate-specific antigen velocity. Although the findings of Sato et al. [10] regarding radical prostatectomy cases suggest that TZ PCa generally has better clinical outcomes compared to PZ cancers, tumors located in other prostate zones may have a higher propensity to metastasize, significantly impacting patient survival and quality of life. As far as we know, no study has investigated prostate tumors' distinct locations and patterns of spread to understand the metastatic risk for patients.

This study aims to investigate the relationship between anatomic locations of PCa and metastasis risk in a Ghanaian population. By focusing on this specific population, we seek to provide insights that could enhance risk stratification and inform tailored treatment approaches.

## Materials and methods

Study design and site

This hospital-based retrospective observational study was conducted from 2019 to 2023 and included consecutive biopsy-confirmed patients with PCa. The study was carried out at Spectra Health Imaging and Interventional Radiology in Kumasi, Ghana, a renowned and well-resourced major referral center for PCa diagnosis equipped with advanced medical imaging facilities. The facility thus provided the needed diverse and relevant sample for this study.

Study population

This retrospective study included adult patients (≥18 years) with biopsy-confirmed PCa diagnosed between 2019 and 2023. Eligible patients had complete medical records with data on demographic characteristics, clinical history, imaging, and disease outcomes. Patients were excluded if they had incomplete records, a diagnosis of benign prostatic hyperplasia (BPH) or prostatic intraepithelial neoplasia (PIN) without confirmed prostate cancer, a history of other cancers, or had received prior treatment for prostate cancer, including surgery, radiation, hormonal therapy, or chemotherapy. 

Sample size calculation and sampling technique

The sample size was calculated using the Cochran formula, considering the 6.0% prevalence of PCa in Ghana [11], a 95% confidence interval (1.96), and a 0.05 margin of error. The minimum sample size calculated was 87, but 120 consecutive eligible cases were included to improve statistical power.

Ethical approval

Ethical approval for the study was obtained from the Committee on Human Research, Publication, and Ethics at Kwame Nkrumah University of Science and Technology (CHRPE/RC/028/20). Patients’ data were deidentified, ensuring anonymity and adherence to relevant guidelines and regulations.

Sociodemographic and clinical data collection

Medical records of study participants were carefully examined and information on sociodemographic factors and clinical factors such as age, PCa type, lesion type, lesion location and dimensions, and incidental findings were extracted. Patient MRIs on picture archiving communication systems were carefully reviewed by two radiologists with 10 and 15 years of experience in prostate MRI. Medical records were also checked for corresponding apparent diffusion coefficient (ADC) values, Gleason scores, and PSA levels for each participant.

Acquisition of images using whole-body diffusion-weighted imaging (WB-DWI)/MRI + biparametric MRI (bpMRI)

All imaging was conducted using a Siemens Magnetom Essenza 1.5 Tesla 16-channel TIM MRI scanner (Siemens Healthineers, Germany). For the prostate examination, the following sequences were used: high-resolution T2 images in the axial, coronal, and sagittal planes and axial DWI (B50-1400) with ADC mapping. The axial DWI examination utilized the Siemens proprietary high-resolution diffusion sequence RESOLVE. Axial T2 and axial diffusion images were fused with color coding for improved visualization. The large flex coil of the scanner was positioned posteriorly at the level of the pelvis, and the body coil was anteriorly positioned at the level of the pelvis. Both coils combined to acquire high-quality images.

**Figure 1:**
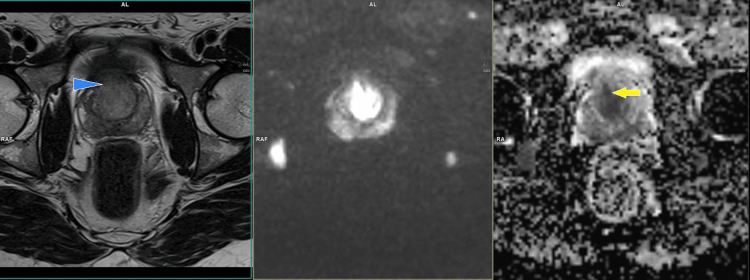
Biparametric prostate MRI of a 50-year-old patient showing diffuse tumor infiltration of the whole gland with the index lesion in the anterior stroma zone, which shows strong restriction in the diffusion sequence. The lesions appear hypointense in the axial T2 sequence (whole arrow) and the axial ADC map (arrowhead).

**Figure 2:**
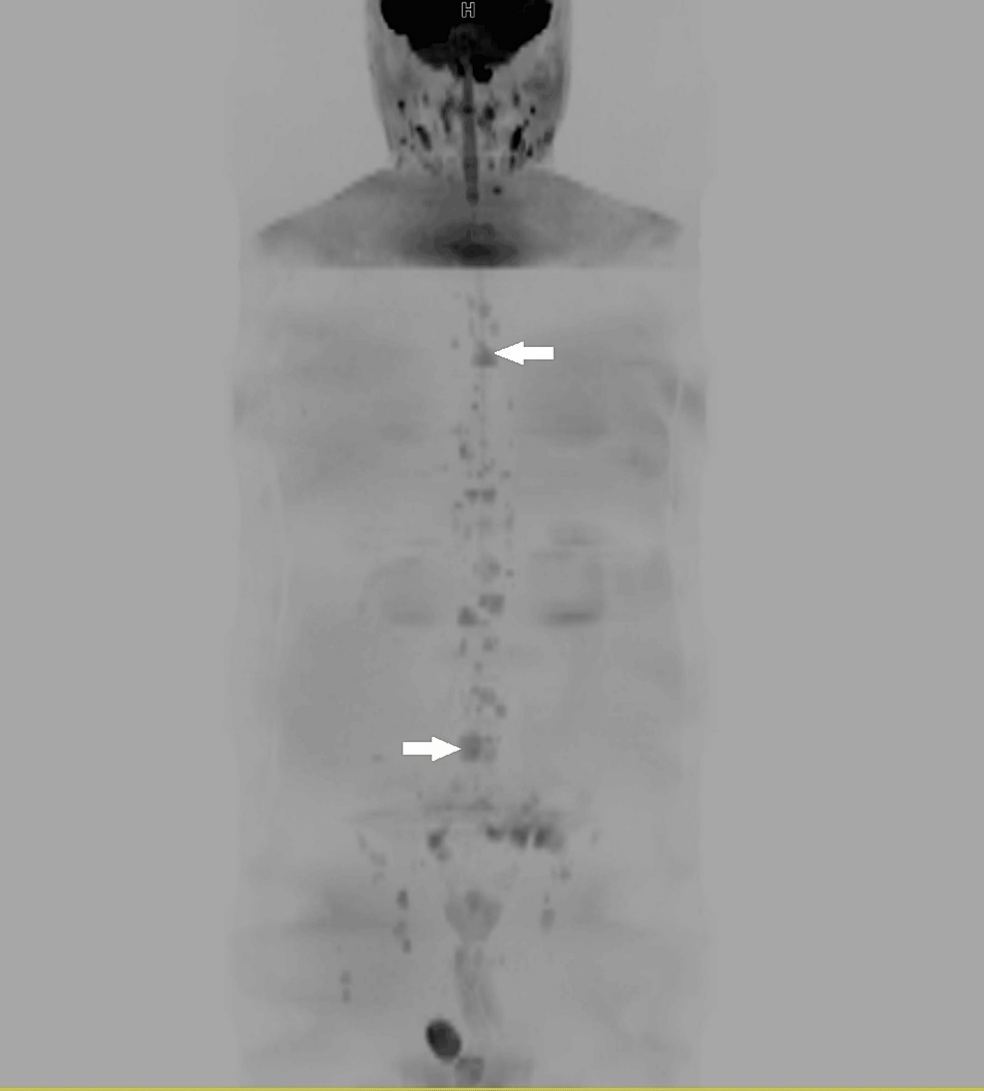
Whole body diffusion-weighted MRI of the same patient with coronal inverted MIP reconstruction showing multiple vertebral metastases (arrows).

In addition, a whole-body diffusion-weighted MRI scan, with an average scan time of 50 minutes, included axial DWI (B50-900) with ADC mapping from the base of the skull to the mid-thigh; T1 and STIR images of the whole spine in the sagittal plane; T2 axial images of the neck, thorax, abdomen, and pelvis; and T2 spin-echo images of the brain. The area of interest was covered using the head and neck coil for the head and neck, the body matrix coil for the thorax and abdomen, and the large flex coil for the pelvis to mid-thigh. High-resolution 3D-maximum intensity projection (MIP) images were reconstructed from the transverse diffusion images into coronal and sagittal planes, after which they were inverted and stitched (Figures 1, 2).

Data management and statistical analyses

The data were collected, entered, cleaned, and coded using Microsoft Excel 2021 (Microsoft Corporation, USA). Statistical analyses were performed using R language for statistical computing version 4.2.3 (R Foundation for Statistical Computing, Vienna, Austria) and IBM SPSS Statistics for Windows, Version 27.0 (released 2020, IBM Corp., Armonk, NY). Categorical variables were presented as frequencies and percentages, and bar charts were used to provide an overview of the data. A logistic regression prediction model was utilized to identify independent predictors of metastasis. A p-value of <0.05 at a 95% CI was considered statistically significant.

## Results

Sociodemographic characteristics of PCa patients

**Figure 3:**
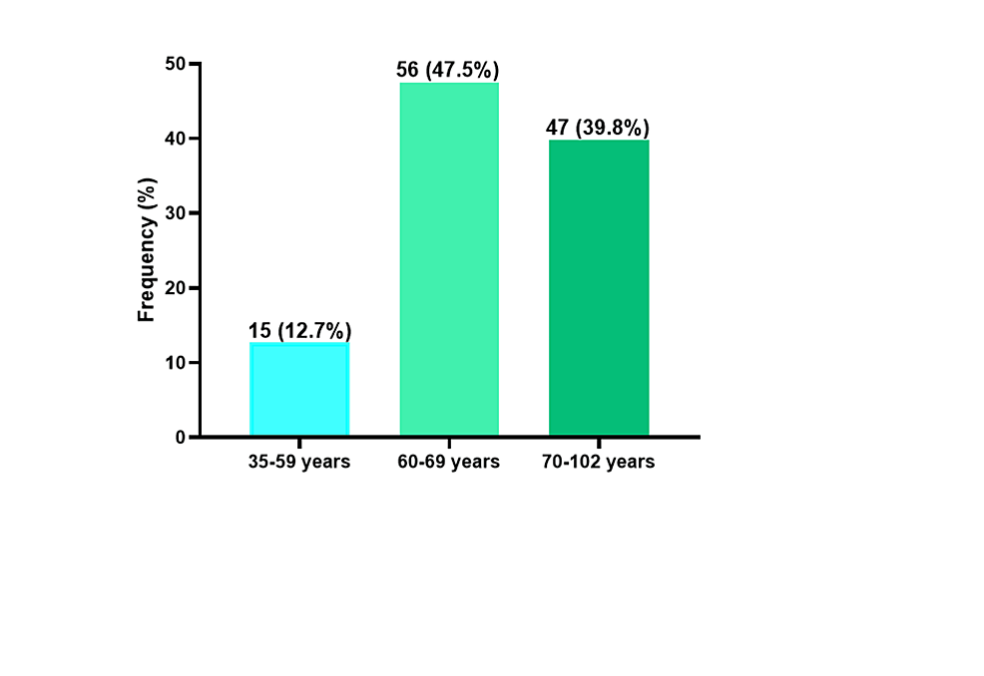
Age distribution of PCa patients

One hundred and twenty patients were included in the study. The prevalence of PCa was more predominant among those aged 60-69 years (47.5%), followed by those aged 70-102 years (39.8%) and the 35-59-year age group (12.7%) (Figure 3).

Pattern of lesion localization among PCa patients

**Figure 4:**
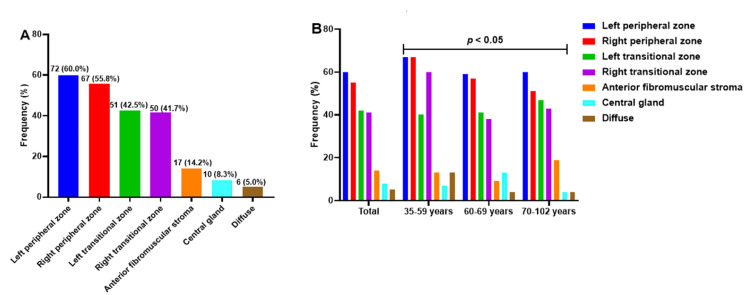
Patterns of lesion localization among PCa patients (A), lesion locations stratified by age (B)

The PCa lesions exhibited a multifocal growth pattern, involving multiple anatomical zones within the prostate gland. The peripheral zones were most commonly affected, with lesions identified in 60.0% of patients in the left peripheral zone and 55.8% in the right peripheral zone. Notably, the transitional zones were also involved, with cancer detected in 42.5% of patients in the left transitional zone and 41.7% in the right transitional zone. In addition, a proportion of lesions were at the anterior fibromuscular stroma (14.2%), central gland (8.3%), or diffuse lesions (5.0%). There were slight differences in the distribution of the lesion localization across different age groups (p < 0.05) (Figure 4).

Metastasis and extracapsular extension among PCa patients

**Figure 5:**
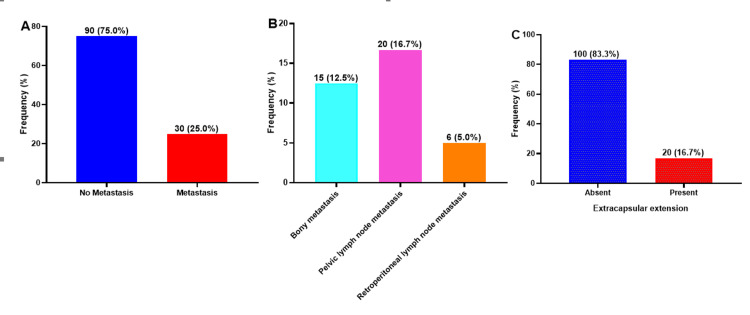
Prevalence (A) and types of metastases (B), including extracapsular extension (C), among prostate cancer patients

We observed that 30 out of the 120 patients had metastasis, representing a prevalence of 25.0%. Pelvic lymph node metastasis (16.7%) was predominant, followed by bony metastasis (12.5%) and retroperitoneal lymph node metastasis (5.0%) (Figure 5A, 5B). As shown in Figure 5C, 20 (16.7%) patients had an extracapsular extension and 83.3% did not.

Distribution of lesion localization as a risk of metastasis and extracapsular extension

In a univariate logistic regression prediction model, lesions in the anterior fibromuscular stroma (crude odds ratio (cOR): 3.27, 95% confidence interval (CI): 1.13-9.47, p = 0.029), central gland (cOR: 5.38, 95% CI: 1.40-20.60, p = 0.014), and diffuse lesions (cOR: 6.78, 95% CI: 1.17-30.07, p = 0.032) were significantly associated with three, five, and six times increased odds of PCa metastasis, respectively. After adjusting for possible confounders, lesions in the anterior fibromuscular stroma emerged as a marginally independent predictor of metastasis among patients with PCa (adjusted odds ratio (aOR): 28.14, 95% CI: 0.96-822.46, p = 0.053). Diffuse lesions (cOR: 5.71, 95% CI: 1.06-30.65, p = 0.042) were significantly associated with a six times increased likelihood of extracapsular extension. In addition, increasing the lesion length (cOR: 1.85, 95% CI: 1.20-2.86, p = 0.005) and breadth (cOR: 1.66, 95% CI: 1.04-2.65, p = 0.033) were significantly associated with two times increased likelihood each of extracapsular extension among patients with PCa. After adjusting for possible confounders, increasing lesion length remained a significant predictor of extracapsular extension (aOR: 5.18, 95% CI: 1.15-23.35, p = 0.032) (Table 1).

**Table 1:** Distribution of lesion localization as a risk of metastasis and extracapsular extension cOR: crude odds ratio, aOR: adjusted odds ratio, CI: confidence interval

Variable	Metastasis (n = 30)	cOR (95% CI)	p-value	aOR (95% CI)	p-value
Lesion length (π ±SD)	2.63 ±1.74	1.39 (0.98-1.98)	0.066	3.37 (0.87-13.10)	0.080
Lesion breadth (π ±SD)	1.79 ±1.24	1.32 (0.87-2.02)	0.196	0.27 (0.05-1.36)	0.112
Lesion type					
Single	11 (36.7)	1.00	-	1.00	-
Double	2 (6.7)	0.32 (0.06-1.58)	0.160	0.19 (0.01-5.73)	0.336
Multiple	17 (56.7)	1.34 (0.55-3.27)	0.517	0.18 (0.01-2.53)	0.202
Location of lesion					
Right peripheral zone	15 (50.0)	0.73 (0.32-1.67)	0.458	-	-
Left peripheral zone	15 (50.0)	0.58 (0.25-1.33)	0.199	0.48 (0.07-3.21)	0.446
Right transitional zone	14 (46.7)	1.31 (0.57-3.02)	0.522	-	-
Left transitional zone	16 (53.3)	1.80 (0.78-4.13)	0.168	0.78 (0.08-7.96)	0.830
Anterior fibromuscular stroma	8 (26.7)	3.27 (1.13-9.47)	0.029	28.14 (0.96-822.46)	0.053
Central gland	6 (20.0)	5.38 (1.40-20.60)	0.014	12.97 (0.30-570.65)	0.185
Diffuse	4 (13.3)	6.78 (1.17-30.07)	0.032	0.09 (0.00-7944.42)	0.674
	Extracapsular Extension (n=20)	cOR (95% CI)	p-value	aOR (95% CI)	p-value
Lesion length (π ±SD)	3.29 ±2.30	1.85 (1.20-2.86)	0.005	5.18 (1.15-23.35)	0.032
Lesion breadth (π ±SD)	2.16 ±1.97	1.66 (1.04-2.65)	0.033	0.19 (0.03-1.23)	0.082
Lesion type					
Single	9 (45.0)	1.00	-	1.00	-
Double	1 (5.0)	0.19 (0.02-1.65)	0.133	0.00 (0.00-inf)	0.998
Multiple	10 (50.0)	0.86 (0.32-2.36)	0.775	0.29 (0.02-5.02)	0.394
Location of lesion					
Right peripheral zone	12 (60.0)	1.23 (0.46-3.26)	0.681	-	-
Left peripheral zone	8 (40.0)	0.38 (0.14-1.00)	0.051	0.38 (0.03-4.86)	0.458
Right transitional zone	6 (30.0)	0.55 (0.19-1.54)	0.251	-	-
Left transitional zone	8 (40.0)	0.88 (0.33-2.35)	0.804	0.46 (0.03-7.15)	0.575
Anterior fibromuscular stroma	5 (25.0)	2.44 (0.75-7.94)	0.137	49.09 (0.55-4379.22)	0.089
Central gland	3 (15.0)	2.35 (0.55-9.97)	0.249	-	-
Diffuse	3 (15.0)	5.71 (1.06-30.65)	0.042	0.02 (0.00-7.60)	0.195

Distribution of lesion localization and risk of specific metastasis

In a univariate logistics regression prediction model, lesions found in the anterior fibromuscular stroma (cOR: 3.88, 95% CI: 1.13-13.26; p = 0.031) and diffuse lesions (cOR: 18.73, 95% CI: 3.07-114.16; p = 0.001) were significantly associated with a four and 19 times increased likelihood of bony metastasis. Increasing the lesion length (cOR: 1.61, 95% CI: 1.09-2.37; p = 0.018) was significantly associated with a two times increased likelihood of pelvic lymph node metastasis. Moreover, lesions found in the central gland (cOR: 6.63, 95% CI: 1.05-41.85; p = 0.044), increasing lesion length (cOR: 2.45, 95% CI: 1.30-4.61; p = 0.006) and lesion breadth (cOR: 2.17, 95% CI: 1.20-3.94; p = 0.010) were significantly associated with a six, two, and two times increased likelihood of retroperitoneal lymph node metastasis, respectively (Table 2).

**Table 2:** Distribution of lesion localization and risk of specific metastasis cOR: crude odds ratio, ADC: apparent diffusion coefficient, PSA: prostate-specific antigen. p-values < 0.05 and presented in bold are statistically significant.

	Bony metastasis		Pelvic lymph node metastasis		Retroperitoneal lymph node metastasis	
Variable	cOR (95% CI)	p-value	cOR (95% CI)	p-value	cOR (95% CI)	p-value
Lesion length (π ±SD)	1.42 (0.94-2.13)	0.093	1.61 (1.09-2.37)	0.018	2.45 (1.30-4.61)	0.006
Lesion breadth (π ±SD)	1.40 (0.85-2.33)	0.188	1.56 (0.99-2.45)	0.055	2.17 (1.20-3.94)	0.010
Lesion type						
Single	1		1		1	-
Double	0.50 (0.05-4.77)	0.547	0.17 (0.02-1.43)	0.103	0.68 (0.07-6.99)	0.748
Multiple	2.22 (0.65-7.64)	0.205	0.67 (0.24-1.82)	0.426	0.52 (0.08-3.23)	0.479
Location of lesion						
Right peripheral zone	0.66 (0.22-1.94)	0.447	0.75 (0.29-1.97)	0.566	0.78 (0.15-4.04)	0.768
Left peripheral zone	0.73 (0.25-2.17)	0.574	0.48 (0.18-1.27)	0.139	0.65 (0.13-3.38)	0.610
Right transitional zone	1.26 (0.43-3.74)	0.675	1.18 (0.45-3.10)	0.741	0.27 (0.03-2.34)	0.233
Left transitional zone	2.25 (0.75-6.79)	0.150	1.13 (0.43-2.97)	0.804	1.38 (0.27-7.11)	0.704
Anterior fibromuscular stroma	3.88 (1.13-13.26)	0.031	2.44 (0.75-7.94)	0.137	3.30 (0.56-19.61)	0.189
Central gland	3.50 (0.80-15.37)	0.097	3.92 (0.99-15.44)	0.051	6.63 (1.05-41.85)	0.044
Diffuse	18.73 (3.07-114.16)	0.001	1.00 (0.11-9.05)	> 0.999	4.36 (0.43-44.66)	0.215

## Discussion

The prostate is anatomically classified into three zones: peripheral (sometimes referred to as the prostate proper), transitional, and central, supported by a non-glandular anterior fibromuscular stroma [12]. In this study, we aimed to investigate the correlation between the location of lesions and the spread of PCa in African men. The majority of PCa lesions were found in the left (60.0%) and right peripheral zones (55.8%), followed by the left (42.5%) and right transitional zones (41.7%). These findings are consistent with the established understanding that the peripheral zone, the largest area within the prostate, is the primary site for most PCa cases [13], with 75% prevalence manifesting predominantly at the dorsal and dorsolateral sides [14]. Similar to our study, the transitional zone accounts for only 20% of apparent PCas [14]. This study reinforces these global trends and extends the understanding of zonal predispositions to the Ghanaian population, highlighting the peripheral zone's susceptibility to PCa development [13,15].

In this study, lesions found in the anterior fibromuscular stroma, central gland, and diffused areas were significantly associated with three, five, and six times increased odds of PCa metastasis, respectively. Consistent with previous research, tumors involving the central zone are linked to more aggressive disease courses, characterized by higher prostate-specific antigen (PSA) values, Gleason scores, extracapsular extension rates, and seminal vesicle invasion rates [6].

Like previous studies, tumors of the anterior prostate account for approximately 14-20% of all PCas [16,17]. Our multivariate logistics regression model identified lesions in the anterior fibromuscular stroma as a marginally independent predictor of metastasis and specifically bone in patients with PCa, suggesting more aggressive cancer behavior in this zone. No study has established a connection between lesions in the anterior fibromuscular region and metastases. Positive anterior lymphofatty tissue lymph nodes with regional spread of cancer have been studied [18,19]. However, whether this has an anatomical connection with the anterior fibromuscular stroma of the prostate remains largely unexplained.

Furthermore, this study found that lesions with a diffuse growth pattern were associated with a higher likelihood of bone metastases in the African cohort. According to Popiolek et al., patients with non-palpable diffuse tumors have nearly two-fold higher mortality and progression rates than those with TO1 or palpable tumors [20]. It is also important to note that their study revealed an average time of 9.2 years until metastasis development. In our current study, localized and metastatic diseases were identified in a single examination, indicating that patients in our cohort presented at late and advanced stages of the disease. These findings underscore the need for improved early detection and disease monitoring strategies tailored to African populations, where PCa often presents at more advanced stages. Prioritizing the recognition of high-risk anatomical patterns, such as diffuse lesions, may help guide personalized risk assessment and management for these patients. These findings highlight the importance of considering lesion location, particularly in high-risk anatomical zones, such as the anterior fibromuscular stroma, central gland, and diffused lesions, when assessing metastatic risk and planning personalized management strategies for PCa patients, especially in African populations where the disease burden is disproportionately high.

In summary, this study represents a significant step forward in advancing our understanding of the complex interplay between PCa lesion locations and their metastatic potential. The results emphasize the need for targeted diagnostic and treatment strategies based on lesion location to optimize PCa management in this population. Identifying high-risk anatomical zones for PCa lesions can help guide clinicians in tailoring screening, surveillance, and therapeutic approaches for patients. Moreover, the heterogeneous nature of PCa across different anatomical zones, as demonstrated in this study, underscores the importance of region-specific research efforts to better understand zonal predispositions.

While our findings are promising, it is important to acknowledge the limitations inherent in our study. First, the sample size, while providing valuable insights into the African population, may not be large enough to fully capture the nuances of zonal predispositions and metastatic patterns. In addition, the study relied on retrospective clinical and radiological data, which may be subject to variability in diagnostic techniques, reporting, and documentation practices across different healthcare settings. Incorporating standardized prospective data collection protocols could help minimize potential inconsistencies and biases. Despite these limitations, this study offers important contributions to the understanding of PCa in African men, highlighting the need for further research and the development of tailored diagnostic and management strategies for this high-risk population.

## Conclusions

This study provides valuable insights into the anatomical localization patterns and metastatic risk of PCa lesions in the Ghanaian population. The findings emphasize the importance of considering lesion location in risk assessment and management strategies, particularly the high-risk zones identified, such as the anterior fibromuscular stroma. Integrating these insights into clinical practice can contribute to more personalized and effective PCa care, ultimately reducing the substantial burden of the disease in African communities. However, it is important to conduct region-specific research to understand the risk of metastasis based on PCa zonal predispositions in diverse populations.
